# Complications following reverse total shoulder arthroplasty for proximal humeral fractures: a systematic review

**DOI:** 10.1016/j.xrrt.2024.08.007

**Published:** 2024-08-29

**Authors:** Easton J. Bents, Javier Ardebol, Mathew Noble, Lisa Galasso, Patrick J. Denard, Mariano E. Menendez

**Affiliations:** a ATSU-SOMA, Mesa, AZ, USA; b Oregon Shoulder Institute, Medford, OR, USA; c UC Davis Department of Orthopaedic Surgery, Oregon Shoulder Institute, Medford, OR, USA

**Keywords:** Reverse total shoulder arthroplasty, rTSA, Proximal humerus fracture, Complications, Systematic review, Revision rate

## Abstract

**Background:**

Reverse total shoulder arthroplasty (rTSA) is an increasingly common procedure for proximal humerus fractures (PHFs), but postoperative complications have not been well characterized. The purpose of this systematic review is to assess complications and revision rates following rTSA in the management of PHFs as documented in current literature.

**Methods:**

A systematic review was performed on primary rTSA for PHFs. In adherence to Preferred Reporting Items for Systematic Reviews and Meta-Analyses guidelines, a systematic search was conducted between September and December of 2023, utilizing the databases PubMed, Scopus, and the Cochrane Library to include relevant articles from any period. A total of 102 studies were identified for inclusion after initial screening of 3800 query results. Extracted data from each article included the publishing journal, level of evidence, study design, number of patients, mean age, age range, percent female, mean follow-up, specific postoperative complications, total complications, complication rate, number of revisions, and revision rate. Overall complication rates were determined by dividing total complications by total patients and multiplying by 100, which also applied to revision rates.

**Results:**

Among 10,797 primary rTSA cases for PHFs identified from 102 studies, the mean age of patients was 75.6 ± 3.6 years, and 82.6% of patients were female. The average follow-up was 38.9 ± 21.0 months. The overall complication rate was 7.0% (754/10,797) with a range of 0% to 46.9%. The most common postoperative complications were prosthetic instability/dislocation (2.3%, 244/10,797; range: 0%-7%) and infection (1.2%, 131/10,797; range: 0%-2.5%). Periprosthetic fracture (0.8%), nerve injury (0.3%), and other complications were also documented. The overall revision rate was 3.3% (355/10,797; range: 0%-28%).

**Conclusion:**

This systematic review summarizes the most common postoperative complications of rTSA for PHFs. The most frequent complication was prosthetic instability/dislocation, followed by infection. While the average total complication and revision rates were relatively low, there was wide variability, with some studies reporting rates as high as 46% and 28%, respectively.

Proximal humerus fractures (PHFs) are the third most common fracture in the elderly.^5^^,^^25^ These fractures occur more often in women and may lead to significant pain and loss of function. Reverse total shoulder arthroplasty (rTSA) has gained increasing acceptance as the mainstay of surgical intervention for PHFs in older patients due to more predictable outcomes over other surgical methods such as open reduction and internal fixation and hemiarthroplasty (HA).^6^^,^^17^^,^^28^^,^^48^^,^^49^^,^^66^^,^^114^^,^^122^^,^^129^ The adoption of rTSA for PHF saw a remarkable surge of 406% from 2005 to 2012, and in 2013, rTSA surpassed HA as the preferred surgical intervention for patients over 65 year old.^31^^,^^51^^,^^121^

Complication rates following rTSA for PHF have varied widely among the current published literature.^21^^,^^32^^,^^37^^,^^42^^,^^78^,^81^,^86^,^93^,^106^ Complications following rTSA for PHFs are mostly derived from relatively small cohorts.^21^^,^^32^^,^^37^^,^^42^^,^^78^^,^^81^^,^^86^^,^^93^^,^^106^ Improved characterization of complications associated with rTSA for PHFs would be of value for patient counseling, setting expectations, and ultimately improving outcomes.

The purpose of this systematic review was to assess complications, as well as revision rates, following rTSA in the management of PHFs as documented in current literature.

## Methods

### Search strategy

This systematic review was performed in line with Preferred Reporting Items for Systematic Reviews and Meta-Analyses guidelines.^79^ PubMed, Scopus, and the Cochrane Library were systematically searched for relevant articles. This search encompassed articles from any period and was conducted between September and December of 2023. To maximize the sensitivity of the search strategy, the following search terms including, “reverse”, “shoulder”, “arthroplasty”, “fracture”, “humerus”, and “proximal” were combined with the Boolean operators, “AND” or “OR”. The final search strategy included the following search terms: (“reverse” AND “shoulder” AND “arthroplasty” AND “Fracture”). The search was limited to English-language articles or articles with English translation.

### Study selection

Table I shows the inclusion and exclusion criteria. Predefined eligibility criteria were clinical trials and observational studies (cohort studies, comparative studies, and case series) that reported on complications after primary rTSA for PHF only. All studies, regardless of level of evidence, were included if they explicitly reported complications after primary rTSA for PHF.^96^ We included studies with a sample size of at least 5 patients and a minimum or mean of at least 1-year follow-up. If a study included a cohort of patients who met the study criteria, but another cohort did not, the study was included and only the data for the patients who met our study criteria was extracted and recorded. Studies missing age or follow-up range were included if the study reported the standard deviation for age or follow-up. Studies with the following criteria were excluded: (1) meta-analyses, systematic reviews, conference abstracts, committee posters, studies on animals, biomechanical reports, tumoral studies, technical notes, letters to editors, instructional course, case reports, conference abstracts, cadaveric studies, and expert-opinion studies; (2) articles that did not clearly report the occurrence and specific nature of complications; (3) studies reporting complications without direct assignment to primary rTSA for PHF; (4) pooled or combined data (ie, primary and revision, rTSA, and HA) that could not be stratified; and (5) studies with a narrow focus solely on a single complication.

**Table I:** Inclusion and exclusion criteria.

Inclusion criteria	
Databases	Pubmed, SCOPUS, Cochrane
Diagnosis	Proximal humeral fracture
Type of surgery	Primary reverse shoulder arthroplasty
Minimum follow-up (mean or min)	≥12 months
Sample size	≥5
Data reported	Complications
Exclusion criteria	
Type of study	Meta-analyses, systematic reviews, conference abstracts, committee posters, studies on animals, biomechanical reports, tumoral studies, technical notes, letters to editors, instructional course, case reports, conference abstracts, cadaveric studies, and expert-opinion studies.
Assessment	Unclear occurrence/rate and/or nature of complication; studies reporting complications without direct assignment to rTSA for PHF; pooled or combined date with other procedures (ie, hemiarthroplasty, ORIF, revision);

*rTSA*, reverse total shoulder arthroplasty; *PHF*, proximal humerus fracture.

### Literature search

The literature search was conducted by one author (E.J.B). Search results were screened first by title, then by abstract, and last by full-text review. Conflict was resolved by discussion with a second reviewer (M.E.M.) to determine inclusion. Relevant data was extracted into a custom Excel spreadsheet, including author, journal, publication year, level of evidence, study design, sample size, patient age, sex, follow-up, postoperative complications, and revisions. Postoperative complications were classified as instability/dislocation, superficial and deep infection, periprosthetic fracture, nerve injury, acromial stress fracture, glenoid fracture, complex regional pain syndrome and reflex sympathetic dystrophy, hematoma, component failure, and delayed wound healing. If other postoperative complications were reported, they were recorded as “Other”. This study did not consider radiographic outcomes such as tuberosity nonunion/malunion, scapular notching, heterotopic ossifications, or component loosening.

### Statistical analysis

Descriptive statistics were used to summarize all collected information from the studies because inferential statistics were not possible. Categorical data was reported as frequency (percentage), and continuous data was reported as mean (range). The overall complication rate was calculated by dividing the total number of complications by the total number of cases and multiplied by 100. This calculation was also used to determine the revision rate. Statistical analysis was performed using Microsoft Excel (Microsoft Excel 2024; Microsoft Corp., Redmond, WA, USA).

## Results

A total of 102 studies were identified for inclusion after database query of 3800 query results. Author, journal, study design, level of evidence, number of patients, sex, age, and follow-up were recorded and are presented in Table II. A total of 10,797 patients received rTSA for PHF as their primary surgery. The mean age was 75.6 ± 3.6 years (range, 27-102), and 82.6% (8918/10,797) of patients were female. The average follow-up was 38.9 ± 21.0 months.

**Table II:** Details of demographic data, including study design and level of evidence of the analyzed studies.

Author	Journal	Year	Level of evidence	Study design	Patients (n)	Mean age	Range (age)	SD (±)	Female (%)	Mean follow-up (months)	Follow-up range (months
Lopiz et al^72^	Journal of Shoulder and Elbow Surgery	2019	1	Prospective randomized control trial	29	82.0		3.2	86.2	12.0	
Rivera et al^99^	Journal of Shoulder and Elbow Surgery	2023	4	Retrospective case series	400	72.0	60-91		87.5	12.0	
Fraser et al^36^	Journal of Bone and Joint Surgery	2020	1	Randomized controlled trial	64	75.7	65-86	6.1	92.2	12.0	3-24
Imiolczyk et al^56^	Journal of Clinical Medicine	2022	3	Retrospective cohort study	27	73.4	56-91	7.8	77.0	50.0	12-142
Jonsson et al^59^	Journal of Shoulder and Elbow Surgery	2021	1	Randomized controlled trial	41	80.4		4.5	95.0	28.8	
Viswanathan et al^128^	Cureus	2023	4	Retrospective case series	34	76.0	61-91		86.0	61.0	36-110
Gallinet et al^37^	Orthopaedics & Traumatology: Surgery and Research	2019	4	Retrospective observational study	898	79.0	46-98		82.0	28.0	12-60
Crespo et al^26^	Journal of Shoulder and Elbow Surgery	2021	3	Retrospective cohort comparison	108	75.3		7.2	86.1	49.5	
Lignel et al^70^	Orthopaedics & Traumatology: Surgery & Research	2018	4	Retrospective multicenter study	513	78.0	65-95		89.9	55.0	
Raiss et al^94^	Journal of Bone and Joint Surgery	2016	4	Retrospective multicenter study	42	68.0	27-83		71.4	48.0	24-156
Kramer et al^64^	BMC Musculoskeletal Disorders	2022	3	Retrospective case-control	51	74.0	58-90	6	84.3	27.0	
Chun et al^20^	Journal of Bone and Joint Surgery	2017	3	Retrospective cohort design	38	80.4	73-89		86.8	36.9	
Weber et al^130^	European Journal of Orthopaedic Surgery and Traumatology	2023	3	Retrospective cohort design	82	70.0		9.8	69.5	48.0	
Luciani et al^75^	Musculoskeletal Surgery	2022	3	Retrospective comparative study	22	75.5		5.6	91.0	33.4	
Chalmers et al^18^	Journal of Shoulder and Elbow Surgery	2014	3	Retrospective cohort study	9	77.0		6	78.0	14.4	
Zafra et al^132^	The Bone and Joint Journal	2014	3	Prospective multicenter study	35	69.0	46-83		80.0	51.0	24-99
Holschen et al^53^	Archives of Orthopaedic and Trauma Surgery	2022	3	Retrospective comparative study	58	78.5			91.3	35.0	24-58
Panagopoulos et al^90^	Journal of Shoulder and Elbow Surgery	2021	3	Retrospective cohort comparison	92	75.6	39-93		75.0	48.0	24-146
Grubhofer et al^49^	Journal of Shoulder and Elbow Surgery	2016	4	Case series	52	77.0	58-89		88.0	35.0	12-90
Formaini et al^34^	Journal of Shoulder and Elbow Surgery	2015	4	Case series	25	77.0	63-88		68.0	17.0	
Siebenburgern et al^118^	Geriatric Orthopaedic Surgery and Rehabilitation	2021	3	Comparative cohort study	76	77.1		7.1	83.0	22.2	
Garofalo et al^41^	Journal of Orthopaedic Surgery and Research	2015	4	Case series	98	76.2	61-90		63.2	27.0	24-32
Boileau et al^8^	Journal of Shoulder and Elbow Surgery	2019	3	Retrospective cohort design	38	80.0	70-88	4	92.0	36.0	24-59
Dezfuli et al^30^	Journal of Shoulder and Elbow Surgery	2016	3	Retrospective cohort design	13	78.0	66-78		85.0	34.0	24-36
Schwarz et al^112^	BMC Musculoskeletal Disorders	2021	3	Retrospective observational study	42	73.6	60-89	6.8		41.0	24-134
Sabah et al^105^	International Orthopedics	2021	4	Retrospective case series	422	79.0	46-97		83.0	50.0	12-108
Santana et al^107^	Acta Orthopaedica et Traumatologica Turcica	2019	4	Retrospective study	9	79.1		7	78.0	54.7	24-108
Ross et al^101^	Journal of Shoulder and Elbow Surgery	2015	4	Case series	29	79.0	67-90		87.0	54.8	25-107
Grassi et al^46^	Journal of Shoulder and Elbow Surgery	2014	3	Retrospective clinical study	15	75.0	70-83		100.0	22.0	12-46
Raiss et al^95^	The Bone and Joint Journal	2014	4	Retrospective multicenter study	32	68.0	48-83		87.5	48.0	24-144
Shannon et al^117^	Journal of Shoulder and Elbow Surgery	2016	3	Retrospective cohort design	18	75.0	60-88		78.0	36.0	24-72
Sebastia-Forcada et al^113^	Journal of Shoulder and Elbow Surgery	2014	1	Randomized controlled trial	31	74.7	70-85		87.1	29.4	24-49
Lopiz et al^73^	International Orthopedics	2016	3	Retrospective study	42	81.7	76-88		81.0	32.6	
Simovitch et al^120^	Journal of Orthopaedic Trauma	2019	4	Retrospective cohort study	55	77.0	65-87		69.1	33.7	24-62
Garofalo et al^39^	Orthopaedics & Traumatology: Surgery & Research	2016	3	Retrospective case series	26	68.5	63-75		69.2	32.0	24-38
Roberson et al^100^	Journal of Shoulder and Elbow Surgery	2017	3	Retrospective cohort design	20	71.0	52-88		95.0	53.0	
Youn et al^131^	Journal of Shoulder and Elbow Surgery	2016	4	Case series	20	76.5	62-87		90.0	36.0	30-94
Lenarz et al^69^	Clinical Orthopaedics and Related Research	2011	3	Retrospective observational study	30	77.0	65-94		90.0	23.0	12-36
Bufquin et al^12^	Journal of Bone and Joint Surgery	2007	4	Prospective case series	43	68.0	65-97		95.3	22.0	6-58
Tuphe et al^125^	European Journal of Orthopaedic Surgery and Traumatology	2023	3	Control-case study	94	77.0	63-97	8	89.4	45.0	24-88
Cuff et al^28^	Journal of Bone and Joint Surgery	2013	2	Prospective comparative clinical study	24	74.8	70-86		58.3	30.0	24-48
Giardella et al^45^	Muscles, Ligaments, and Tendons Journal	2017	4	Case series	21	77.2	65-91	6.4	85.7	40.0	12-66
Lo et al^71^	Journal of Shoulder and Elbow Surgery	2021	4	Case series	60	67.0	47-85		71.7	21.0	10-46
Jeong et al^58^	Archives of Orthopaedic and Trauma Surgery	2019	4	Case series	45	80.0	65-92	7	82.2	40.8	18-72
Baudi et al^6^	Musculoskeletal Surgery	2014	4	Retrospective comparative study	25	77.0	missing		86.8	27.5	12.0-64.0
Gunst et al^50^	International Orthopedics	2021	3	Retrospective cohort study	28	77.0	60-93	8	85.0	14.4	12.0-25.0
Sebastia-Forcada et al^114^	The Bone and Joint Journal	2020	2	Prospective cohort study	67	73.5	52-85		77.6	100.8	60.0-121.0
Uzer et al^126^	Journal of Shoulder and Elbow Surgery	2017	3	Retrospective cohort design	33	73.5	65-82		63.6	16.8	12.0-25.0
Klug et al^63^	Journal of Shoulder and Elbow Surgery	2020	3	Retrospective cohort comparison	30	73.9		6.7	83.3	38.0	12.0-50.0
Boyer et al^11^	European Journal of Orthopaedic Surgery and Traumatology	2017	2	Prospective clinical evaluation	65	78.0	66-91			15.0	6.0-41.0
Siebenburger et al^119^	Geriatric Orthopaedic Surgery and Rehabilitation	2021	3	Retrospective cohort comparison	60	78.9		39.3	76.7	39.5	27.0-68.0
Chivot et al^19^	Journal of Shoulder and Elbow Surgery	2019	3	Retrospective cohort design	28	77.0	70-92		78.6	31.8	24-.052.0
Russo et al^103^	Musculoskeletal Surgery	2015	3	Retrospective cohort study	95	75.0	62-95		84.2	60.0	12.0-108.0
Cazeneuve et al^15^	Orthopaedics & Traumatology: Surgery and Research	2011	4	Retrospective study	35	75.0	58-92		94.2	30.0	12.0-204.0
Gallinet et al^38^	Orthopaedics & Traumatology: Surgery & Research	2009	4	Retrospective comparative study	16	74.0	58-84		81.2	12.4	6.0-18.0
Obert et al^84^	Orthopaedics & Traumatology: Surgery & Research	2016	4	Retrospective and prospective multicenter study	73	78.8	60-91		90.3	25.2	5.0-62.0
Joseph et al^61^	Journal of Shoulder and Elbow Arthroplasty	2022	3	Retrospective cohort study	28	74.4		8.5	89.2	165.5	53.0-371.0
Gasbarro et al^44^	Geriatric Orthopaedic Surgery and Rehabilitation	2019	3	Retrospective cohort study	46	73.4	66-84			37.2	12-60
Ostergaard et al^88^	Journal of Shoulder and Elbow Surgery	2022	3	Retrospective cohort comparison	74	71.6		8.1	79.7	55.6	36-85
Claro et al^22^	BMC Geriatrics	2023	2	Prospective comparative study	112	78.6		5.8	86.6	52.0	
Barbosa et al^3^	Geriatric Orthopaedic Surgery and Rehabilitation	2020	3	Retrospective cohort study	35	73.5	65-81		88.6	38.3	24-68
Jonusas et al^60^	Archives of Orthopedic and Trauma Surgery	2017	3	Retrospective cohort study	27	67.5	55-85	7.3	74.0	45.0	39-48
Ortmaier et a^87^	International Orthopedics	2015	4	Case series	25	73.0	65-89		88.0	32.7	24-45
Garofalo et al^40^	Injury	2015	4	Retrospective cohort study	22	77.2	65-84		77.3	24	1-24
Fischer et al^33^	Archives of Orthopedic and Trauma Surgery	2023	3	Prospective cohort study	56	81.0	53-91		95.0	24	1.5-24
Barger et al^4^	Injury	2021	3	Retrospective cohort study	114	73.5		8.5		15.6	12-82
Critchley et al^27^	Journal of Shoulder and Elbow Surgery	2020	3	Retrospective cohort study	3049	75.4	32-102	8.7	84.1	complex	1-120
Fortane et al^35^	Orthopeadics and Traumatology: Surgery and Rehabilitation	2020	3	Retrospective cohort study	34	78.0	63-91	5.7	79.0	18.0	1-40
Izquierdo-Fernandez et al^57^	Journal of Orthopaedics and Traumatology	2021	3	Controlled cohort study	32	74.1	65-87		90.6	84	12-84
Garrigues et al^43^	Orthopedics	2012	3	Retrospective comparative study	10	80.5	67-97			43.2	16-96
Bonnevialle et al^10^	Orthopeadics and Traumatology: Surgery and Research	2016	3	Multicenter retrospective study	41	78.0	60-88	5	90.0	39.0	25-63
Torrens et al^124^	Journal of Orthopaedic Surgery	2018	3	Retrospective cohort study	41	77.9	62-90	6.3	75.6	29.0	24-37
Tian et al^123^	Orthopaedic Surgery	2020	4	Case series	43	72.0	66-78		72.1	10.9	6-16
Haws et al^52^	Journal of Orthopaedic Trauma	2023	3	Retrospective cohort study	26	76.8		7.3	100.0	26.4	12-60
Schmalzl et al^110^	European Journal of Orthopaedic Surgery & Traumatology	2020	3	Retrospective cohort study	64	76.0		7	86.0	22.0	
Schmalzl et al^111^	BMC Musculoskeletal Disorders	2020	4	Retrospectivecase series	38	77.0	62-97	8	87.0	34.0	
Rossi et al^102^	Journal of Shoulder and Elbow Surgery	2022	3	Retrospective cohort comparison	67	74.0	65-84		66.0	41.0	24-72
Sasanuma et al^108^	Journal of Shoulder and Elbow Surgery International	2020	3	Retrospective cohort study	41	78.0	70-88	6.4	88.1	29.0	24-49
Cazeneuve et al^16^	Orthopeadics and Traumatology: Surgery and Research	2009	4	Therapeutic retrospective study.	30	75.0	58-92		92.7	78.0	12-168
Cappellari et al^14^	Injury	2022	3	Retrospective cohort study	91	76.0	65-87		85.0	46.0	24-120
Lanzetti et al^67^	Clinical Orthopaedics and Related Research	2023	2	Therapeutic study	72	76.0		2.9	56.0	53.0	24-72
Garofalo et al^39^	Journal of Clinical Medicine	2023	3	Retrospective case-control	68	72.3	65-84		70.0	80.4	60-94
Iacobellis et al^55^	Musculoskelatal Surgery	2015	3	Retrospective cohort study	33	76.6	54-85		84.8	42.3	10-121
Bonnevialle et al^9^	European Journal of Orthopedic Surgery and Traumatology	2019	3	Retrospective multicenter study	150	77.0	59-93		93.0	59.0	24-180
Reuther et al^98^	Journal of Orthopedic Trauma	2019	4	Case series	81	78.5	58.5-90.9	6.5	88.9	24.8	12-77
Repetto et al^97^	Musculoskeletal Surgery	2017	3	Therapeutic series	27	71.2	65-80	7.5	85.0	41.7	12-78
Greiner et al^47^	Journal of Shoulder and Elbow Surgery	2015	1	Randomized controlled trial	34	75.4	66-88		64.7	22.0	
Zhang et al^133^	Orthopedics	2023	3	Retrospective cohort study	153	69.5		8.3	75.8	50.3	24-85
Paszicsnyek et al^92^	Journal of Clinical Medicine	2023	3	Matched cohort analysis	134	69.0		11	75.0	58.0	
Blaas et al^7^	Shoulder and Elbow	2023	3	Retrospective comparative study	53	74.4		7.8	90.6	20.0	
Claro et al^23^	Journal of Shoulder and Elbow Surgery	2022	3	Retrospective comparative study	45	80.0		5.2	86.7	64.2	
Muller et al^80^	Archives of Orthopedic and Trauma Surgery	2022	3	Retrospective cohort study	40	76.8		6.8	85.0	33.5	
Dao Trong et al^29^	Geriatric Orthopaedic Surgery and Rehabilitation	2022	3	Retrospective cohort study	62	87.0	83-98		90.0	37.0	3-148
Ohl et al^85^	Journal of Shoulder and Elbow Surgery	2018	3	Retrospective multicenter cohort study	420	77.7	48-97	7.7	86.4	28.0	12-60
Russo et al^104^	Joints	2015	4	Case series	75	67.0	36-95		64.1	84.0	
Colasanti et al^24^	Journal of Shoulder and Elbow Surgery	2023	3	Retrospective comparative study	322	72.0		9	82.6	47.1	24-138
Ott et al^89^	Seminars in Arthroplasty: Journal of Shoulder and Elbow Surgery	2021	3	Retrospective comparative study	30	79.0	69-94	6.6	87.0	37.3	26-57
Candela et al^13^	Seminars in Arthroplasty: Journal of Shoulder and Elbow Surgery	2021	3	Retrospective comparative study	73	76.4	66-82		78.1	32.0	24-66
Kuhlmann et al^65^	Seminars in Arthroplasty: Journal of Shoulder and Elbow Surgery	2020	3	Retrospective comparative study	144	74.0		7.8	83.3	48.5	
Verdano et al^127^	Musculoskeletal Surgery	2018	3	Retrospective comparative study	32	77.4	67-92		75.0	14.3	
Sasanuma et al^109^	Journal of Orthopaedic Science	2023	3	Retrospective cohort study	35	79.2	65-92		94.3	35.0	24-81
Marin et al^77^	Journal of Orthopaedic Science	2023	3	Retrospective cohort study	90	74.0	51-86		78.0	34.0	12-67

*SD*, standard deviation.

The occurrence and complication rates are presented in Table III. Eleven studies met the inclusion but specifically reported zero complications for primary rTSA for PHFs, leaving ninety-one studies that reported complications for rTSA following PHF. The overall complication rate was 7.0% (n = 754), with a range of 0% to 46.9%. The most common complication was prosthetic instability or dislocation with a rate of 2.3% (n = 244; range, 0%-7%). The second most common complication was infection with a rate of 1.2% (n = 131; range 0%-2.5%). The rates of periprosthetic fracture was 0.8% (n = 89; range, 0%-2.4%); 0.7% (n = 74; range, 0%-1.6%) for nerve injury; 0.3% (n = 27; range, 0%-0.8%) for acromial stress fracture; 0.3% (n = 28; range 0%-3.3%) for glenoid fracture; 0.2% (n = 24; range, 0%-0.8%) for pain, complex regional pain syndrome, and reflex sympathetic dystrophy; 0.2% (n = 23; range, 0%-0.4%) for hematoma; 0.1% (n = 15; range, 0%-1.2%) for component failure; and 0.01% (n = 1; range, 0%-0.1%) for delayed wound healing. The overall revision rate was 3.3% (n = 355), with a range of 0% to 28%.

**Table III:** Summary of postoperative complications following RSA for PHFs.

Complications	No. of complications	% of all cases (n = 10,797)	% of all complications (n = 754)	Range (n)	Range (%)
Instability/dislocation	244	2.3	32.4	0-53	0-7.0
Infection	131	1.2	17.4	0-19	0-2.5
Periprosthetic fracture	89	0.8	11.8	0-18	0-2.4
Nerve injury	74	0.7	9.8	0-12	0-1.6
Acromial stress fracture	27	0.3	3.6	0-6	0-0.8
Glenoid fracture	28	0.3	3.7	0-25	0-3.3
Pain/CRPS/RSD	24	0.2	3.2	0-6	0-0.8
Hematoma	23	0.2	3.1	0-3	0-0.4
Component failure	15	0.1	2.0	0-9	0-1.2
Delayed wound healing	1	0.0	0.1	0-1	0-0.1
Other∗	98	0.9	13.0	0-28	0-3.7

*CRPS*, chronic regional pain syndrome; *RSD*, reflex sympathetic dystrophy.

∗ Other complications reported as followed: respiratory tract infection, UTI, Acute delirium, acute kidney injury, medical complications-exacerbation of lung and/or heart disease, DVT, confusion, stiffness, malpositioning of glenoid component, sensibility disturbance, thromboembolic complications, tubercle impingement, Adhesions with intractable stiffness, Separation of anterior muscular flap, hinderance on stem screw, Osteolysis, capsulitis, phlebitis, problems with locking screw removal, arthrofibrosis, Seroma, Sepsis, Subclavian artery damage, impingement, cuff rupture, hypersensitivity reaction, anemia, seroma, death due to renal failure, keloid, vertical bone fissuring, and deltoid muscle damage.

## Discussion

The purpose of this systematic review was to assess the complications following rTSA in the management of PHFs as documented in current literature. Additionally, the secondary outcome of this review was to document revision rates. The primary finding of this study showed that the overall complication rate of rTSA for PHFs was 7.0% but rates varied widely with some studies showing rates as high as 46%. Prosthetic instability/dislocation was the most common complication followed by infection. The overall revision rate was 3.3%, with rates ranging from 0% to 28%. To date, our study is the largest review of literature detailing postoperative complications following rTSA for PHF, covering 102 studies with 10,797 cases.

The most common complication following primary rTSA for PHF in this review was instability or dislocation with a rate of 2.3%. Gallinet et al demonstrated similar findings in a retrospective review of rTSA for PHFs showing that instability was the most common complication, occurring at a rate of 2.5% (22/898).^37^ The authors also reported that excision of the tuberosity was the only factor significantly associated with a higher risk of instability (*P* < .0001). Furthermore, several studies have suggested that patients undergoing rTSA for PHFs have a higher risk of instability and/or dislocation with advanced age and female sex.^2^^,^^54^^,^^116^ In regards to other indications for rTSA, Ahlquist et al, showed in their retrospective review of 15,678 patients that patients undergoing rTSA for PHFs more likely to develop instability at 90 days (odds ratio [OR] 2.26, 95% confidence interval [CI] 1.65-3.04, *P* < .001), 1 year (OR 2.30, 95% CI 1.74-3, *P* < .001) 2 years (OR 2.02, 95% CI 1.53-2.62, *P* < .001) than patients with degenerative joint disease (DJD), which included glenohumeral arthritis, cuff tear arthopathy, post-traumatic arthritis, and inflammatory arthropathy, to develop instability.^1^ They showed that at 90 days postop, the instability rate after rTSA for DJD and PHFs to be 2.3% (340/14,515) and 4.5% (52/1163) (*P* value < .001), respectively; at 1 year postop to be 3.1% (443/14,515) and 5.8% (67/1163) (*P* value ≤ .001), respectively; and at 2 years postop to be 3.5% (504/14,515) and 5.9% (68/1163) (*P* value ≤ .001), respectively. Confirming the similar trend of instability rates between specific surgical indications for rTSA, Paras et al, in a systematic review and meta-analysis, found that in 77 studies for elective rTSA including rotator cuff tear arthropathy, primary glenohumeral arthritis, and massive irreparable rotator cuff tears, vs. 40 studies for rTSA for PHFs, the rates of instability/dislocation were similar, with an instability rate of 0.9% in the elective group (*P* value < .001) and an instability rate of 1.4% in the PHF group (*P* value = .241).^91^ However, the authors did not include the number of patients in each group or the mean follow-up times of each group. While the investigation by Paras et al and Ahlquist et al highlights the consistency in instability rates across diverse surgical indications for rTSA, exploration into the impact of implant designs on stability contributes to optimizing outcomes in shoulder arthroplasty procedures. Implant designs have evolved to allow glenosphere lateralization and humeral distalization to improve stability.^83^ It’s important to note that our study did not specifically consider prosthesis design or surgeon technique and did not make comparisons to other indications for rTSA for PHFs.

The rate of infection after primary rTSA for acute PHFs has been reported to range between 0.2% and 2.4%.^1^^,^^68^^,^^115^ Consistent with the literature, our review showed that infection occurred at a rate of 1.2%; however, our report did not distinguish between early vs. delayed rTSA for PHFs, fracture type, or implant type. Other studies have shown no difference with infection rates between indications for rTSA.^1^^,^^26^ Ahlquist et al, showed in their retrospective review 15,678 patients no significant difference in incidence of infection when performed for PHFs compared to nontraumatic indications.^1^ Alhquist et al reported that at 90 days postop, the infection rate after rTSA for DJD and PHFs to be 0.1% (16/14,515) and 0.2% (2/1163) (*P* value = .59), respectively; at 1 year postop to be 0.3% (45/14,515) and 0.3% (4/1163) (*P* value = .99), respectively; and at 2 years postop to be 0.4% (55/14,515) and 0.3% (4/1163) (*P* value = .99), respectively. They concluded that there was no difference in prosthetic joint infection rates between rTSA for PHF vs. DJD.

In this review, postoperative periprosthetic fracture occurred at a rate of 0.8%. Alhquist et al, showed that at 90 days postoperatively, the periprosthetic fracture rate after rTSA for DJD and PHFs to be 0.3% (41/14,515) and 0.9% (10/1163) (*P* value < .001), respectively; at 1 year postop to be 0.6% (93/14,515) and 1.7% (20/1163) (*P* value ≤ .001), respectively; and at 2 years postop to be 0.9% (135/14,515) and 1.3% (27/1163) (*P* value ≤ .001), respectively.^1^ They concluded that rTSA for PHF is associated with a higher rate of subsequent periprosthetic fracture than other indications for rTSA. They also demonstrated that rTSA for PHF increases the risk for periprosthetic fracture at 90 days (OR = 3.66, 95% CI = 1.69-7.3), 1 year (OR = 3.07, 95 % CI = 1.81-4.98), and at 2 years (OR = 2.57, 95% CI = 1.64-3.88). Our study, along with corroborating evidence from Alhquist et al,^1^ underscore the heightened risk of periprosthetic fracture associated with rTSA for PHFs compared to other indications.

In the past decade, there have only been a handful of studies specifically investigating postoperative complications of rTSA for PHFs. In a recent review comparing delayed vs. acute treatment with rTSA for PHF, Lu et al reported an overall complication rate of 11.7% (risk ratio: 0.55; *P* = .015) for acute rTSA for PHFs when compared to delayed rTSA for PHFs.^74^ Conversely, Nelson et al conducted a systematic review of five studies, comparing 104 patients who underwent primary rTSA for PHFs with 147 individuals who underwent salvage rTSA following failed open reduction and internal fixation for PHFs. Their findings revealed significantly lower complication rates: 4.8% (5/104) for primary rTSA compared to 18.7% (28/150) for salvage procedures. Moreover, they reported that the odds of experiencing a complication were 78% lower among patients undergoing primary rTSA for PHFs rather than as a salvage procedure (*P* = .002).^82^ In contrast to other indications for rTSA, Malik et al demonstrated in a comparative study of outcomes that patients who underwent shoulder arthroplasty for PHFs faced a higher risk of surgical complications compared to those undergoing shoulder arthroplasty for osteoarthritis.^76^ Specifically, among 8283 patients who underwent either anatomic or rTSA for PHFs vs. 667 patients treated for osteoarthritis, the risk of surgical complications was significantly elevated (OR = 2.44, 95% CI = 1.31-4.55, *P* value = .005). They proposed that due to the nonelective nature of shoulder arthroplasty for PHFs that the immunologic and physiologic response to trauma may predispose the patient to more postoperative complications. The overall complication rate in our study was 7%.

The current literature shows less variability when it comes to overall revision rates. Our study showed an overall revision rate of 3.3%. In the previously mentioned systematic review of acute vs delayed rTSA for PHFs, Lu et al, showed a revision rate of 1.8% (203/11,289) for acute treatment and 4.4% (497/11,289) for delayed treatment (Risk ratio = 0.44; 95% CI = 0.34-0.59; *P* < .001).^74^ They concluded that time to operation was valuable in decreasing the revision rate. Keller et al, showed in a retrospective review of 21 patients who were treated with rTSA for PHFs and 27 patients who were treated with rTSA for rotator cuff tear arthropathy that at 2-year follow-up there was no difference in revision surgery rates between PHF and rotator cuff tear arthropathy patients (10% vs. 15%, *P* value = .58).^62^ The authors concluded that their study provided validation and support for the recent shift in preference for employing rTSA in the management of PHFs. Furthermore, Critchley et al showed in a retrospective cohort comparison using a large database that among 3049 individuals undergoing primary rTSA for PHFs and 2897 patients undergoing HA for PHFs, the cumulative revision rate at 9 years was lower for those who underwent rTSA (7.0%; 95% CI = 4.8-10.1) compared to HA (11.7%; 95% CI = 10.3-13.2).^27^

This systematic review has several limitations. These limitations include those inherent to systematic reviews, such as the inclusion of numerous retrospective studies that may introduce both individual and compounded reporting biases. Our study does not consider the timing of rTSA performed following PHF, such as acute vs. delayed rTSA for PHFs, nor did it account for the presence of comorbidities. Additionally, not every article commented on the complications analyzed in this report. Furthermore, this study did not consider radiographic outcomes such as tuberosity nonunion/malunion, scapular notching, heterotopic ossifications, or component loosening. Finally, the heterogeneity of the articles, whether it was based on level of evidence, type of fracture, approach, implant design, or specific technique used, could not be fully accounted for.

## Conclusion

This systematic review summarizes the most common postoperative complications of rTSA for PHFs. The most frequent complication was prosthetic instability/dislocation, followed by infection. While the average total complication and revision rates were relatively low, there was wide variability, with some studies reporting rates as high as 46% and 28%, respectively.

## Disclaimers:

Funding: No funding was disclosed by the authors.

Conflicts of interest: Dr. Denard is a consultant for and receives royalties from Arthrex, Inc. Dr. Menendez is a consultant for Arthrex, Inc. The other authors, their immediate families, and any research foundation with which they are affiliated have not received any financial payments or other benefits from any commercial entity related to the subject of this article.
